# Pain a Blessing

**Published:** 1860-01

**Authors:** 


### PAIN A BLESSING.

Pain is the sleepless sentinel, always at the outposts, announcing on the instant the first approach in the distance, of the great enemy disease; and that half humanity dies scores of years "before the time," is because the faithful warning goes all unheeded. Suppose, for example, a man gets "boozy," and falls asleep, if fire gave no pain, he might wake up next morning minus a foot, or nose, or with a hole in a head, all empty. A plain case.

